# Report of Seven Cases of Tracheotomy

**Published:** 1878-05

**Authors:** E. W. Lee

**Affiliations:** Chicago


### REPORT OF SEVEN CASES OF TRACHEOTOMY IN DIPHTHERIA AND CROUP, WITH TWO RECOVERIES.

By E. W. Lee, M. D., Chicago.

*Case I.—Diphtheria.*

At 2 a. m., August 29, 1877, I visited Nannie Keegan, aged 3 years and 10 months, a healthy looking well nourished child. The parents stated that she had been sick three days. Her condition at this time was as follows: extreme dyspnœa, the head thrown back, the epigastrium retracted, and all the muscles assisting in respiration called into full play; the skin hot, pulse 160, and countenance livid.

An examination of the throat revealed diphtheritic exudation on the fauces, velum, and tonsils, which appeared to extend deeply into these parts. A physician had been in attendance for a couple of days, employing the usual remedies without preventing the progress of the disease, and had finally expressed his opinion that further medication was useless, and the only hope of saving the child was in the operation of tracheotomy. As I fully coincided in this opinion, I immediately proceeded to operate, assisted by Drs. Hutchinson, Taliaferro, and Landis. After some delay, owing to hemorrhage, the tube was successfully introduced, chloroform being the anæsthetic employed. Immediate relief ensued, and the patient rallied finely. The pulse fell to 130, with a general alleviation of the distressing symptoms. The child passed a comfortable night, partaking freely of nourishment, and on my visit next morning, was in a favorable condition ; pulse 120, and the respiration accelerated, but easy. About two hours after, I was summoned in haste, and on arrival found the child struggling with extreme dyspnœa, evidently dying. There was profuse expectoration of a sanious mucus, and crepitant râles were distinctly heard over both lungs. The tube was immediately removed, but without benefit. The child died within an hour.

The remarkable features of this case were the sudden accession of these unfavorable symptoms, and the rapidity with which they progressed.

*Case II.—Diphtheria.*

John Phalon, Irish, æt. 4, a stout, healthy looking boy, had been complaining of sore throat for three or four days. On my visit, Oct. 11, 1877, a.m., I found him playing about the house, apparently not in much distress. He had croupy cough, which had begun the day before, and his breathing was now becoming stridulous. On examination, found the tonsils and soft palate covered with a well-marked diphtheritic exudation. The pulse was from 120 to 130, the skin moist and respiration accelerated. The disease was evidently extending into the larynx. As none of these symptoms existed in an aggravated form, the case was deemed a suitable one for constitutional treatment. Accordingly the patient was put upon a solution containing iron, quinine, and chlorate of potassium. The disease made no apparent progress for the remainder of that day, and the next morning (12th) there seemed to be some improvement, which condition was maintained throughout that day.

Oct. 13th, a.m.—Condition about the same. In the afternoon the breathing became somewhat labored, and in the evening I was sent for, with the information that the child was suffocating. On my arrival, found him sleeping very quietly ; stridor had disappeared, the pulse had fallen, and also, the respiration.

In explanation, the parents stated that he had vomited a large piece of tough, whitish-looking substance, which he held between his teeth for a moment, but swallowed on their attempting to remove it. Relief from the distressing symptoms was immediate, and the child fell into a quiet sleep.

Oct. 14th, a.m.—Child again playing about the house. Medicine continued at increased intervals. 15th.—All evidences of the disease had disappeared, and I ceased my attendance. 16th.—Was again sent for, and found the child in about the same condition as on my first visit. Active treatment was again resumed, and in addition the steam atomizer, with a solution of carbolic acid and chlorate of. potassium and glycerine, was employed. During the day the child's condition gradually became worse. Bronchial râles were heard over both lungs. 17th, a.m.—No improvement; p.m.—Growing worse. 18th, a.m.—Greatly distressed; urgent dyspnœa; head thrown back, epigastrium retracted, countenance livid, the pulse rapid and feeble, asphyxia imminent. Tracheotomy was now advised as the only procedure affording any hope of relief.

After considerable hesitation, the parents reluctantly consented to the operation, which was then rapidly performed, the patient under chloroform. Drs. Bridge, Taliaferro and Landis assisted. Immediate relief was experienced on the introduction of the tube. Brandy, quinine and beef tea ordered. The child rested well during the remainder of the day, but the tube required frequent clearing of a thick, tenacious membrane.

Oct. 19th, 8 a.m.—Passed a comfortable night; pulse 133, respiration 36. The tube required constant attention. Treatment continued. 9 a.m.—Vomited for the first time the brandy and beef tea, which were given together. 11:20.—Condition so much improved that he sat up in bed and played with toys. He retained his medicine and nourishment. 12 m.—Coughed up a piece of membrane. 4 p.m.—Pulse 142, respirations 24 and noiseless; tongue moist and clammy. 6 p.m.—Had several fits of coughing, and a larger piece of membrane was expelled; also some tenacious puriform mucus. 9:15 p.m.—Coughed up a piece of tube-cast, $\frac{3}{4}$ inch in length, perfect in shape; also a considerable quantity of puriform mucus, with some shreds of membrane.

From this time, this muco-purulent discharge increased in quantity.

Oct. 20th, 8 a.m.—Passed a restless night. Increase of mucus in bronchi. Brandy had been given at lessened intervals. Stomach very irritable. Notwithstanding these unfavorable symptoms the child rallied so that the pulse fell to 134, and respiration 36. This condition, however, lasted only an hour. The case now assumed a decidedly unpromising aspect, the pulse and respiration slowly rising. In 3 or 4 hours the breathing became so labored that the tube was removed, with the hope of affording relief. This failed, as the disease had evidently extended to the small divisions of the bronchi. Owing to the supporting treatment which had been zealously carried out, the patient's strength remained good till the last. He died by asphyxia, at 2 p.m., struggling violently.

*Case III.—Membranous Croup.*

Oct. 22, 5 a. m.—I visited Clara Nolan, Irish, æt. 7, spare habit, extremely nervous temperament, and poorly nourished. Had been complaining of "croup" for about two weeks, though playing about up to the afternoon previous. She was then taken with paroxysms of croupal dyspnœa. A physician was called, who administered an emetic of Turpeth mineral and employed the steam atomizer. These measures afforded temporary relief. At 4 a. m. the child's respiration became so labored that asphyxia was imminent. I was hastily summoned, and found the child nearly moribund; the head retracted, the epigastrum sunken, struggling violently for breath; pulse 160, resp. 40 to 50, and every evidence of severe nervous shock. On examining the throat there was no membrane visible. Hopeless as the case appeared, I determined upon an immediate operation. Chloroform was administered and the operation hastily performed—Drs. Taliaferro and Landis assisting. After the introduction of the tube, we supposed, for a few moments, that the child was dead, as she ceased breathing. Artificial breathing, however, restored her. The reaction in this case was very violent; pulse rising to 168, resp. 70, temp. 104°. Brandy, beef tea and quinine were freely administered.

Oct. 23.—A general amelioration of the symptoms occurred.

Oct. 24.—4 p. m. Increased obstruction of breathing. Being present I removed the tube, when a large piece of membrane was expelled by violent coughing. This, on examination, was found to be a little over a line in thickness, about 1¼ inch in length, and ½ inch in breadth. The breathing immediately became easier and the tube was reinserted.

From this time the child slowly convalesced, though at times the pulse and respiration were accelerated to an alarming degree. This, however, we came to recognize as due to nervous influences. On the sixth day the tube was permanently removed, and a wire frame, covered with flannel, which was fitted to the neck to protect the wound, was worn until the latter healed. There were no unfavorable sequelæ and the child ultimately made a complete recovery.

*Case IV.—Membranous Croup.*

Nov. 16, 1877, p. m.—I visited Matthew Curran, Irish, æt. 4, a stout, healthy looking boy. His parents stated that he had been taken 36 hours previously with a croupy cough. On examination found pulse 140 and running up to 160 during the paroxysms of dyspnœa. Breathing was stridulous, and the cough characteristic. Dyspnœa was extreme; the attitude of patient the same as described in the other cases. An emetic of Turpeth mineral was administered, with some relief.

Nov. 10, a. m.—The breathing more laborious; râles quite distinctly heard over the chest, and a general aggravation of the unfavorable symptoms. It was evident that further medication was useless. On consultation it was deemed advisable to perform tracheotomy, which was accordingly done, with the assistance of Drs. Bridge and Landis. The dyspnœa was immediately relieved and the pulse fell to 130. The respiration, however, continued rapid. Beef tea, brandy and quinine were freely administered. The case apparently progressed favorably for 48 hours, when the respiration, which had continued rapid, became more labored. The tube was removed several times without affording any relief. The child struggled through the night, and died from exhaustion at 6 a. m. of the 20th.

*Case V.—Membranous Croup.*

Nov. 23, 1877, p. m., I visited Eddie Nolan, Irish, æt 4, in consultation with Dr. Landis. The child had been taken with croupal symptoms 5 days previously, and had been treated, principally, with Turpeth mineral, according to Meigs and Pepper's plan, which stayed the progress of the disease for four days, giving hope of ultimate success. On the morning of the 5th day, however, the treatment failed to afford the usual relief, and the child's condition became rapidly worse. On my visit, the patient presented all the unfavorable symptoms detailed in the cases heretofore described. The patient's strength was so much reduced that an operation hardly promised success. The child being a brother of Clara Nolan, case 3, the parents were anxious that the operation be performed. It was accordingly done, Drs. Bridge, Taliaferro and Landis assisting. Notwithstanding the fact that there was not a trace of membrane to be seen in the throat when the trachea was opened, a large quantity of thick, softened membrane, which was blackish in appearance, was expelled through the opening. The operation relieved the dyspnœa for a time, but the respiration remained rapid. The tube had to be removed several times through the night on account of paroxysms of dyspnœa and coughing, when several pieces of discolored membrane were expelled, which always afforded momentary relief. The child was freely stimulated, but without effect, as his powers of absorption were evidently almost *nil.* He gradually sank, and died of exhaustion at 10 a. m. the next morning. During the periods when the tube was removed, membrane could plainly be seen through the opening in the trachea, and several pieces were removed by the forceps.

*Case VI.—Membranous Croup.*

Dec. 6. 1877. I visited Alice Turcell, æt. three and one-half, a remarkably fine and unusually large child for the age, who had been attacked three days previously, with croup. Her parents stated that she was subject to the affection, but that they had always succeeded in "doctoring her out of it." This time, however, the remedies failed. On examination, the child was found to be suffering from dyspnœa, stridulous breathing, and the pulse was 120 and the respiration 40. Patches of membrane were visible on the tonsils and fauces. Râles were heard over the chest. Emetics were used with marked relief, and I began to hope that an operation would not be necessary.

Dec. 7, p. m. Condition much improved. At 2 p. m., the unfavorable symptoms returned with all their former violence, and tracheotomy was now the only hope. At 6 p. m., assisted by Drs. Marshall, Taliaferro, and Meade, I performed the operation. Relief followed the insertion of the tube, and the child passed a very comfortable night.

Dec. 8th, a. m. Pulse 140, resp. 40. No change during the day.

Dec. 9th, a. m. Marked improvement occurred during night. Pulse falling over 20 beats, respiration 15.

Dec. 10th, a. m. No change and it now seemed that a successful issue might reasonably be expected ; p. m., respiration slowly rising and the tube requiring more frequent cleaning. Pulse remained about the same.

Dec. 11th, a. m. Dispnœa became more urgent and the pulse 140. The child's strength remained good throughout the day, but she died from asphyxia, at 5 p. m.

*Case VII.—Membranous Croup.*

March 17, 1878, p. m., I visited Michael O'Rourke, 26 months of age, a well-nourished child of Irish parents, and was informed that he had been seized with symptoms of croup on the previous day. I found him in the following condition : Pulse 144, resp. 42, and stridulous, skin hot and dry, nostrils distended, dyspnœa marked and epigastrium retracted. There was no membrane in sight. Voice whispering. Ordered a solution containing chlorate of potassium and tincture of iron.

March 18, a. m.—I found an aggravation of all the above mentioned symptoms and I became satisfied that an operation would eventually be necessary.

Being satisfied that the operation is generally performed at too late a period to afford much chance of recovery, I advised that it be done in this case while the vital powers of the child remained good.

Accordingly, at 2 p. m., with the assistance of Drs. Bridge and Landis, I proceeded to operate, chloroform being the anæsthetic employed.

On opening the trachea a quantity of softened membrane was expelled. After the insertion of the tube, the child experienced the relief usually obtained in these cases.

The pulse, immediately after operation, was 144, resp. 68, temp. 101° F. Iron, quinine and beef tea were ordered. During the day several pieces of membrane were expelled through the tube. 11 p.m.—Pulse 136, resp. 56. Child restless. 19th, 8 a.m. Pulse 128, resp. 44, temp. $100\frac{3}{4}$°. Passed the night comfortably.

Mar. 19th, 12 p.m.—Pulse 132, resp. 44. During the day several pieces of membrane were expelled. The child partook freely of nourishment and has rested quietly.

Mar. 20th, 8 a.m.—Pulse 128, resp. 45, temp. $100\frac{3}{4}$°. Passed a good night. 7:30 p.m.—Pulse 134, resp. 38, Child in good condition.

Mar. 21st, 8 a.m.—Pulse 112, resp. 46, temp. $99\frac{3}{4}$°.

Mar. 22d, 10 a.m.—Removed the tube, and as it was found that the child could breathe without, it was not replaced.

From this date the little patient made a rapid recovery.

REMARKS.—*The Operation.*—Chloroform was employed in all these cases. I think it better to use an anæsthetic, not only does it facilitate the performance of the operation, but it eases the breathing by relaxing spasm. From the first incision to the exposure of the trachea, need not occupy much time, but, unless in exceptional cases, the trachea should not be opened until all hemorrhage has ceased.

As large a tube as practicable should be used, small tubes are easily obstructed, and require such frequent cleansing, as to needlessly irritate the patient. Silver tubes are best. Rubber tubes lose the proper curve after being in use for a time.

*The time to Operate.*—If this operation be done earlier, the percentage of recoveries will undoubtedly rise; when long delayed, the chances are decidedly against a successful issue. While I think it ought to be done, no matter how desperate the condition, I would advocate an earlier resort to operative interference. Case III proves the possibility of success under the most unfavorable circumstances, as the child was almost moribund at the beginning of the operation. Case VI might, I think, have recovered had the operation been made 24 hours earlier. As it was, the pulse and respiration fell so rapidly, as to lead me to feel almost certain of its recovery. Case VII was the only one where the operation was performed in reasonably good time, and the easy and rapid recovery made, would seem to impress the importance of an earlier operation. The strength was good, there was no need for alcoholic stimulation, and the condition steadily improved from the time the tube was inserted. It was not expected to find such a quantity of membrane in the trachea, as was discovered in this case. The child had shown the first croupal symptom but 48 hours previously, and although the respiration was rapid, it was not labored to a very marked degree. I was satisfied that operative interference would ultimately be necessary, and determined to give the little fellow all the chances of its early performance. My experience of the operation as a last resort, I think justified me in this. From the date of my first operation, I saw 9 cases where I advised its performance. In 2 the parents refused their consent—both died—in 7 I was permitted to act. I have come to the conclusion that when respiration has been difficult for some hours, without abatement, the voice husky, and other unfavorable symptoms present and corresponding, if an operation be in prospect, the longer it is delayed the less chance there is of success. Very often the treatment for the 24 hours previous to the operation, is largely instrumental in procuring an unfavorable result. Vomiting a child every 3 or 4 hours, to remove obstruction, is poor preparation for such an operation.

In case 5, although the breathing was rendered easy, the most powerful stimulation failed to rally the child, and the most that could be claimed, was that the operation rendered death comparatively easy.

*Unity or Duality of Croup and Diphtheria.*—As to the unity or duality of croup and diphtheria, I must leave its discussion in abler hands than mine. I think the facts are in favor of the dual theory.

*Management.*—The temperature of the apartment was kept from 75° to 80° F., and the atmosphere moistened by the use of steam atomizers. The inner tube was frequently cleansed and when that failed to remove the obstruction, a spray of lime water and glycerine was directed into it. Frequently it was found that the patient breathed easier with the inner tube removed, in that case the outer tube was cleansed by means of a *dry* feather gently introduced and turned round so as to catch the obstructing substance. Failure to relieve obstruction always caused the respiration to become more rapid and in time the pulse also, hence the necessity for skilled attendants. The last six cases had constant medical attendance, and I take this opportunity of returning thanks to Drs. Landis and Taliaferro and Mr. Harold Moyer for their assiduous attention in aiding me in watching these little patients. Case III would undoubtedly have terminated fatally had there not been a competent person in attendance, as the obstruction in the tube could not be removed by the ordinary means.

*Treatment.*—The first six cases were operated on as a "last resort" and were all in feeble condition from the prolonged efforts at respiration and in some cases from the treatment. Brandy was freely used, also iron, quinine, beef tea, and milk in some cases. Considerable difficulty was experienced in having these remedies retained, the stomach being in a very irritable condition and the patient averse to taking either medicine or nourishment. In case III, any abatement in the stimulation was followed by a change for the worse in the child's condition. For the first three days, brandy was given three or four times an hour, besides the other nourishment.

